# Tumor-Infiltrating Lymphocytes Predict Extranodal Extension and Prognosis in Regionally Advanced Oral Cavity Cancer

**DOI:** 10.3390/diagnostics15192431

**Published:** 2025-09-24

**Authors:** Mia Lorencin Bulic, Martin Jurlina, Danko Müller, Lada Lijovic, Matija Mamic, Ivica Luksic

**Affiliations:** 1 Department of Maxillofacial and Oral Surgery, University Hospital Dubrava, Avenija Gojka Suska 6, 10000 Zagreb, Croatia; mjurlina@kbd.hr (M.J.); mmamic1@kbd.hr (M.M.); luksic@kbd.hr (I.L.); 2 School of Medicine, University of Zagreb, Salata 3b, 10000 Zagreb, Croatia; dmuller@kbd.hr; 3 Department of Pathology and Cytology, University Hospital Dubrava, Avenija Gojka Suska 6, 10000 Zagreb, Croatia; 4 Department of Anesthesiology, Intensive Care and Pain Management, Sestre Milosrdnice University Hospital Center, 10000 Zagreb, Croatia; l.lijovic@amsterdamumc.nl; 5 Laboratory for Critical Care Computational Intelligence, Department of Intensive Care Medicine, Amsterdam Medical Data Science, Amsterdam Public Health, Amsterdam Cardiovascular Science, Amsterdam Institute for Infection and Immunity, Amsterdam UMC, Vrije Universiteit, 1081 HV Amsterdam, The Netherlands

**Keywords:** oral cavity squamous cell carcinoma, tumor-infiltrating lymphocytes, tumor microenvironment, extranodal extension, prognostic factor

## Abstract

**Background/Objectives:** Oral cavity squamous cell carcinoma (OCSCC) is an aggressive malignancy, often diagnosed at an advanced stage and with stagnant survival outcomes despite advances in surgical and oncologic management. Tumor-infiltrating lymphocytes (TILs) have been explored as potential prognostic markers in many solid tumors; however, their role in OCSCC remains under researched. This study aimed to assess the prognostic value of TILs in a cohort of patients with regionally advanced, p16-negative squamous cell carcinoma of all oral cavity subsites and to evaluate for any correlation of TILs and extranodal extension (ENE). **Methods:** A retrospective study was conducted on 103 consecutive patients treated with comprehensive surgical resection. TILs were quantified using the standardized method proposed by the International Immuno-Oncology Biomarkers Working Group. Statistical analyses evaluated associations with a comprehensive set of independent variables and survival endpoints. **Results**: High stromal infiltration at the invasive margin (>25%) was independently associated with significantly improved overall survival (HR 4.53, *p* = 0.005), disease-specific survival (HR 4.49, *p* = 0.008), and disease-free survival (HR 3.42, *p* = 0.025). Patients with ENE demonstrated lower TILs compared with ENE-negative patients (median 40% vs. 57.5%), a difference that reached statistical significance in both parametric and nonparametric testing (Welch’s *t*-test *p* = 0.032; Mann–Whitney U *p* = 0.030). **Conclusions**: TILs quantified by this standardized method are a reliable, independent prognostic biomarker in regionally advanced OCSCC of all subsites and are also associated with extranodal extension of regional metases. This study gives rationale for consideration of inclusion of TILS into future immunotherapeutic decision-making and further investigations of TIL-ENE association.

## 1. Introduction

Oral cavity squamous cell carcinoma (OCSCC), the most common malignancy in the head and neck region excluding non-melanoma skin cancers, has remained a challenging disease with stagnant survival outcomes despite advances in surgical and oncological treatment [1,2,3]. Advances in radiotherapy techniques, development of new chemotherapy protocols, and the availability of microvascular free flap reconstruction enabling more radical surgical resection have not yet significantly diminished the high mortality and morbidity rates associated with OCSCC, and the 5-year survival rates have reached a plateau over the last several decades [4,5,6].

At the time of diagnosis, tumors are frequently locally advanced, often exhibiting perineural and lymphovascular invasion, worst pattern of invasion and tumor budding, which are all features associated with an increased risk of recurrence and mortality [7,8,9,10]. Even more troubling are the numbers of cases that are regionally advanced at presentation: several studies have established that a significant number of patients undergoing elective neck dissection have histologically confirmed occult micrometastases in regional lymph nodes, with an established extensive adverse effect on prognosis [11]. In the last edition of the American Joint Committee on Cancer (AJCC)’s Cancer Staging Manual, extranodal extension (ENE) has been incorporated in the TNM staging system after overwhelming evidence on the significant adverse effect of ENE on survival outcomes [12,13,14]. However, factors associated with ENE are underexplored, and increasing research on tumor microenvironment has attempted to elucidate the implications of host immune response in disease spread [15].

Although the TNM system is the basis for prognostic prediction, further immunological characterization of OCSCC could enable the development of new prognostic tools and additional immunotherapeutic strategies [16]. Emerging data has highlighted the role of the tumor immune microenvironment, particularly distribution and density of tumor-infiltrating lymphocytes (TILs) in affecting disease course and response to systemic therapy and predicting both. TILs are T-cells that migrate from peripheral circulation into tumor tissue as part of the body’s immune response. Numerous studies on different tumor types have shown the association between higher TIL counts and better survival outcomes [17,18,19]. However, there have simultaneously been some major contradictions: while some studies suggest that lymphocytic infiltration of the tumor tissue represents the body’s beneficial immune defense and is a favorable prognostic factor, some indicate the opposite: that it might promote tumor growth and progression, acting as a negative prognostic factor [20,21,22]. While the prognostic relevance of TILs has been extensively studied in several different malignancies, like breast, lung or laryngeal cancer [23,24,25], its implications in OCSCC remain underexplored. Additionally, unlike many other malignancies, OCSCC is lacking in well-defined molecular targets, with some that show promise, but so far with limited response rates [26].

## 2. Materials and Methods

This is a single-center retrospective study on consecutive patients treated for regionally advanced, p16-negative squamous cell carcinoma of the oral cavity proper. Primary treatment was comprehensive surgical resection of the tumor with neck dissection and immediate free flap reconstruction of the defect as necessary. Surgical treatment was performed in the Department of Maxillofacial and Oral Surgery, University Hospital Dubrava Zagreb, the largest and referral center for oral cavity cancer in Croatia. Patients who had previously been treated for other malignancies in the head and neck region and those who had received neoadjuvant therapy were excluded from the study. Minimum required follow-up for inclusion in the study was 5 years or until the occurrence of death. 

The following patient data was collected from medical records in accordance with the guidelines provided by the AJCC Cancer Staging Manual, 8th Ed.: patient age, sex, ECOG score, tumor sublocalisation within the oral cavity, prognostic stage group according to TNM stratification, the presence or absence of clinical signs of extranodal extension (cENE), tobacco and/or alcohol use, record of depression diagnosis and any adjuvant treatment administered (chemotherapy and/or radiotherapy) [12]. Patients with incomplete data were excluded from the study.

Unlike in squamous cell carcinoma of the oropharynx, where p16 status is an established prognostic marker, the prognostic value of p16 in OCSCC remains unconfirmed. To avoid potential confounding, we restricted our analysis to p16-negative tumors, thereby ensuring a homogeneous cohort in that respect.

Histologic characteristics of the tumor were collected from our database or reevaluated on hematoxylin–eosin (HE)-stained slides: grade of differentiation, depth of invasion (DOI), perineural invasion (PNI), lymphovascular invasion (LVI), the presence or absence of worst pattern of invasion (WPOI-5), resection margins, lymph node yield (LNY), number of lymph nodes positive for metastasis, lymph node ratio (LNR), number of positive lymph node regions, lowest positive region, pathohistological extranodal extension (pENE), number of lymph nodes with ENE, and extent of microscopic ENE in millimeters.

Additionally, tumor tissue was evaluated for the extent of lymphocytic infiltration according to the standardized scoring method for assessing lymphocytic infiltration in solid head and neck tumors, as described and proposed by the International Immuno-Oncology Biomarkers Working Group [27]. As described in the original article, TILs were assessed in two tumor regions—the invasive margin of the tumor and the central part of tumor tissue, and in two compartments of those two regions—stromal and intraepithelial. Therefore, lymphocytic infiltration was quantified as a percentage in four different categories: sTIL (IM) (percentage of stroma occupied by lymphocytes at the invasive margin), sTIL (TC) (percentage of stroma occupied by lymphocytes in the tumor center), iTIL (IM) (percentage of intraepithelial compartment occupied by lymphocytes at the invasive margin) and iTIL (TC) (percentage of intraepithelial compartment occupied by lymphocytes in the tumor center). Stromal TILs were scored as the percentage of stroma occupied by lymphocytes, and intratumoral TILs as the percentage of tumor islands occupied by lymphocytes. Areas of stroma that did not directly relate to the tumor, as well as areas of fibrosis or central necrosis, were excluded from scoring. Both sTILs and iTILs were scored in two regions of each sample—the invasive front and the tumor center. For example, each slide was visually scanned by light microscopy, and the percentage of TILs at the invasive front was estimated separately for the intratumoral part and for the stromal part, and the same was performed for the tumor center. As per the scoring instructions, the focus was not on the “hot-spots”, rather the average of TILs was reported for each area, expressed semiquantitatively as an incremental parameter (for example, 10%, 30%, etc.). To illustrate, a value of 30% sTILs meant that roughly 30% of the stromal area on the slide was occupied by TILs. Each slide was scanned at low magnification first (5x–10x) and then additionally analyzed at higher magnification (20x–40x). Full, untrimmed sections from the resected tumors were used. Inclusion criteria were at least one representative section (4 to 5 μm) available for each case. Additionally, low-quality samples or sections without tumor–stroma interface were excluded from the study.

All slides were independently scored by two investigators blinded to patient identity and outcome. Interobserver agreement for the key variable sTIL (IM) was assessed using the intraclass correlation coefficient (ICC).

ROC analysis was run to define optimal cutoff points for analyzed variables where appropriate. Association was evaluated between age, sex, tumor sublocalisation, stage, grade, PNI, LVI, WPOI-5, and ENE, and sTIL (IM), which is the category that has shown prognostic value in univariable and multivariable analyses across all endpoints. Univariable and multivariable Cox regression analyses evaluated associations between collected variables and overall survival (OS), disease-free survival (DFS) and disease-specific survival (DSS). All variables showing statistical significance in the univariable Cox regression analysis (*p* < 0.05) were considered for entry into the multivariable analysis. Stepwise selection was applied, with variables retained at *p* < 0.05. Collinearity between nodal variables (LNR, number of ENE-positive nodes, and extent of ENE) was assessed; where strong correlation was detected, the variable with the strongest univariable association was initially retained. The final model identified independent predictors that remained significant after adjustment for correlated covariates. Hazard ratios (HR) and 95% confidence intervals (CI) are reported for all analyses. OS, DSS, and DFS were illustrated in Kaplan–Meier survival curves, with significance confirmed by the log-rank test. All statistical analyses were performed using Python’s Scikit-learn 1.7.1, Statsmodels 0.14.4 and Lifelines 0.30.0. *p*-values < 0.05 were considered statistically significant.

This study was approved by the Ethics Committee of University Hospital Dubrava (Ethical approval code: 2020/2807–05, 3 August 2020). The raw data generated in this study is available upon request from the corresponding author. 

## 3. Results

A total of 103 consecutive patients who fit the study criteria were included, classified as AJCC prognostic stage III (9.7%), stage IV.A (45.6%), or stage IV.B (44.7%). As was expected, most patients were male (87.4%) and most tumors were localized in the oral tongue or floor of the mouth (70.9%). Our cohort was overwhelmingly male (87%), which reflects regional epidemiology, with a historically higher alcohol and tobacco consumption in men. The limited number of female patients (*n* = 13) precluded meaningful sex-based subgroup analysis and is a limitation of this study. ECOG performance status was available and collected for all patients: the majority had good functional status, with ECOG 0 in 30 (29.1%) patients, ECOG 1 in 64 (62.1%), and ECOG 2 in 9 (8.7%). No patients were ECOG ≥ 3, as all were deemed suitable candidates for surgical treatment. Ten patients (9.7%) had a depression diagnosis documented, and heavy tobacco and alcohol consumption was documented with 77.6% and 36.9% of patients, respectively. A total of 17 patients (16.5%) showed clinical signs of extranodal extension (cENE) on examination. Also, 17 patients (16.5%) received no adjuvant therapy, while most (80.5%) received adjuvant radiotherapy. Most tumors were well or moderately differentiated (41.7% and 42.7%, respectively), and perineural invasion and worst pattern of invasion were present in most (62.1% and 56.3%, respectively). Lymphocytic infiltration of tumor tissue ranged from 5% to 95%. Interobserver agreement for sTIL (IM) scoring was high, with an ICC of 0.87 (95% CI 0.82–0.91). Cutoff points defined by ROC analysis are shown in Table 1. 

**Figure 1:**
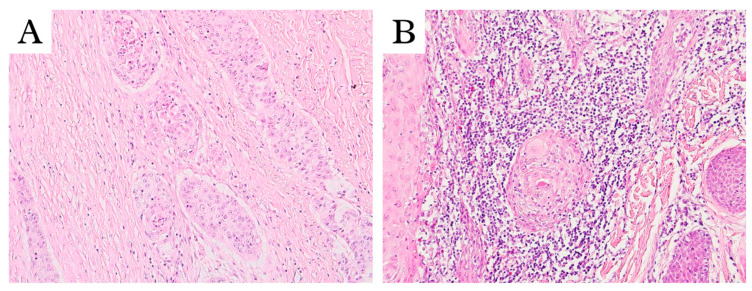
Representative samples of low (≤25%) sTIL (**A**) and high (>25%) sTIL (**B**); 200x magnification.

The cutoff point for TILs that was found most relevant was 25% in the sTIL (IM) compartment. Examples of slides with low and high TIL values are shown in Figure 1.

Patient cohort characteristics distribution with between sTIL (IM) distribution is summarized in Table 2. 

In line with previous research, about half of our regionally advanced patients exhibited extranodal extension (ENE): 51 (49.5%). Patients with ENE had lower sTIL (IM) values compared with ENE-negative patients (median 40% vs. 57.5%), a difference that reached statistical significance in both parametric and nonparametric testing (Welch’s *t*-test *p* = 0.032; Mann–Whitney U p = 0.030) which corresponds to a small-to-moderate effect size (Cohen’s d = −0.43; rank-biserial correlation = 0.25). In ROC analysis, sTIL (IM) discriminated between ENE-positive and ENE-negative patients with an AUC of 0.624 (95% CI 0.511–0.727). The optimal cutoff value regarding this effect was ≤35% sTIL (IM), with a sensitivity of 41.2%, specificity of 80.8%. Logistic regression confirmed the association, with each 10% increase in sTIL (IM) being associated with a reduction in the odds of ENE (OR 0.83, 95% CI 0.70–0.99, *p* = 0.035). Among ENE-positive patients, no significant correlation was observed between sTIL (IM) and the extent of ENE in millimeters (Pearson r = 0.09, *p* = 0.54; Spearman ρ = −0.10, *p* = 0.47). ENE prevalence was 42.2% in WPOI-5 negative cases, and 55.2% in WPOI-5 positive cases. This difference did not reach statistical significance (Fisher’s exact test OR 1.68, *p* = 0.235; χ^2^ *p* = 0.269). In the univariable logistic model, WPOI-5 was not independently associated with ENE (OR 1.68; 95% CI 0.77–3.70; *p* = 0.194).

In the univariable Cox regression analysis, variables that appeared statistically significant across all endpoints (OS, DSS and DFS) were disease stage, LVI, WPOI-5, DOI, resection margin status, LNR, number of lymph nodes with ENE, extent of ENE, and three of the TIL variables: iTIL (IM), sTIL (IM), and sTIL (TC), while iTIL (TC) appeared statistically significant only for DSS. Other collected variables did not show statistical significance in the univariable analysis either. Results of univariable analysis with HR, 95% CI and *p*-values are summarized in Table 3 for all significant variables.

In the multivariable Cox regression analysis, there were two variables that appeared statistically significant across all three endpoints—OS, DSS, and DFS: DOI and sTIL (IM), which is summarized in Table 4 with HR, 95% CI and *p*-values shown.

**Figure 2:**
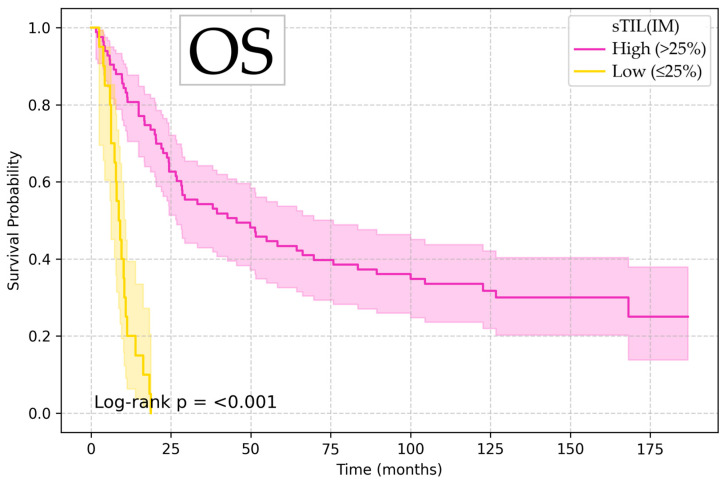
Kaplan–Meier curve for overall survival according to sTIL (IM).

**Figure 3:**
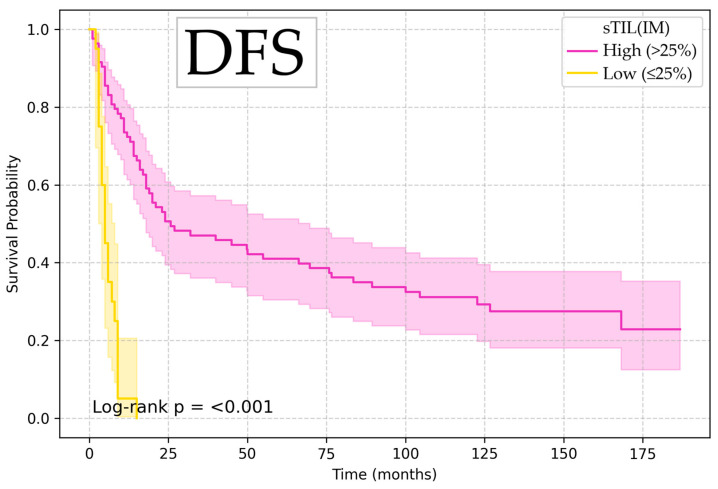
Kaplan–Meier curve for disease-free survival according to sTIL (IM).

**Figure 4:**
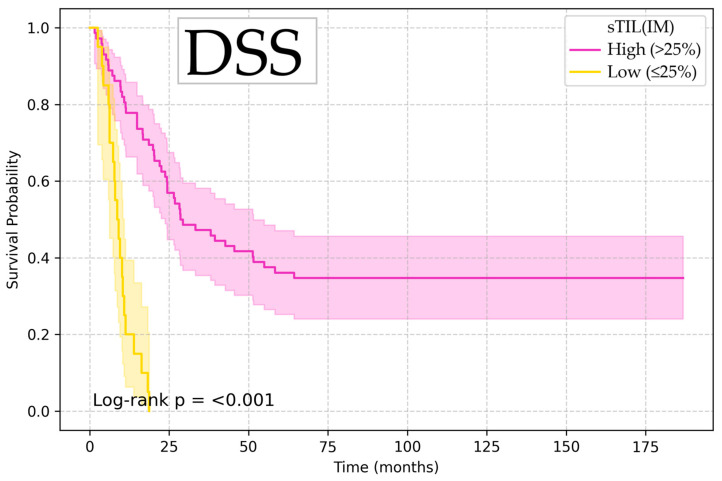
Kaplan–Meier curve for disease-specific survival according to sTIL (IM).

Kaplan–Meier survival analysis showed that low sTIL (IM) values (≤25%) were associated with significantly increased risk of death and disease recurrence (OS HR 4.53, 95% CI 1.57–13.10, *p* = 0.005; DSS HR 4.49, 95% CI 1.48–13.58, *p* = 0.008; and DFS HR 3.42, 95% CI 1.17–9.99, *p* = 0.025), with log-rank test confirming the significant difference between groups, illustrated in Figure 2, Figure 3 and Figure 4.

Although LNR and macroscopic ENE (>2 mm) showed strong prognostic significance in univariable analysis, they did not retain significance in the multivariable analysis, most likely due to collinearity with depth of invasion and other nodal burden variables. This suggests that their prognostic significance in our cohort may not be independent but rather mediated through their association with overall burden of disease. 

## 4. Discussion

In our cohort of 103 consecutive patients, sTIL (IM) have stood out as a clear positive predictor of survival. High scores of sTIL (IM) were consistently associated with a survival advantage across all endpoints, independent of other established adverse tumor features such as LVI, PNI, or WPOI-5 and independent of important treatment factors such as resection margin status, LNR, or adjuvant therapy. 

Our results demonstrated that lower sTIL (IM) value is associated with the presence of ENE in regionally advanced oral cavity squamous cell carcinoma. Although the discriminative performance of sTIL (IM) alone was modest, the consistent direction of effect across comparative testing, ROC analysis, and logistic regression support the relevance of this finding. The magnitude of the difference between ENE-positive and ENE-negative patients was moderate, suggesting that TILs may not be an independent predictor of ENE but rather a complementary biomarker within multiparametric risk assessment. Importantly, the lack of correlation between sTIL (IM) and the measured extent of ENE in millimeters indicates that immune infiltration is more pronouncedly linked to the occurrence of ENE than to its quantitative burden. This aligns with existing evidence that immune competence at the tumor–host interface contributes to the containment of nodal disease, and that attenuated lymphocytic infiltration of the tumor may facilitate the progression of metastasis beyond the lymph node capsule. Taken together, these results address the potential utility of incorporating TIL assessment into composite prognostic frameworks alongside already established histopathological variables such as DOI, PNI, and WPOI-5.

It is worth noting that the ROC-derived sTIL (IM) cutoff associated with ENE (35%) was slightly higher than the threshold that appeared prognostically most significant for survival outcomes (25%). This difference is expected and most likely reflects the differences between endpoints: survival is influenced by multiple tumor- and treatment-related variables beyond ENE. From a clinical perspective, they collectively indicate that reduced immune infiltration below approximately 30% is generally unfavorable. We acknowledge that any numeric threshold derived from a single cohort should be regarded as exploratory and requires external validation in prospective trials before clinical application.

In our cohort, WPOI-5 showed a trend toward association with higher ENE prevalence, but this was not statistically significant and did not relate to the extent of ENE measured in millimeters, suggesting that invasive pattern alone was not sufficient as a stand-alone predictor of ENE in this cohort.

Our findings are consistent with those studies in head and neck cancer that suggest TILs at the tumor–host interface reflect active immune surveillance and tumor containment [28]. The finding that sTIL (IM) carries a stronger prognostic impact than TILs in the tumor center is plausible. The invasive margin is in fact the dynamic tumor–host interface, the area where tumor cells actively invade the surrounding healthy tissue and where immune surveillance mechanisms are expected to be most engaged. A denser lymphocytic infiltrate in this tumor area might signify recognition and containment efforts by the immune system. In contrast, the center of tumor tissue is often affected by hypoxia and necrosis and, therefore, a more immunosuppressive environment, which could limit the migration and activity of immune cells.

An important and notable parallel to our study can be found in the study by Heikkinen et al. on early-stage oral tongue squamous cell carcinoma. This study applied the same standardized scoring method and reported that ≥20% sTIL (IM) was a feature associated with improved survival outcomes [29]. Although our cutoff point was slightly higher (25% vs. their 20%), the consistency in association with prognostic advantage (and at a similar cutoff point) underscores the reproducibility and clinical relevance of sTIL assessment across different OCSCC cohorts. The slightly higher cutoff identified in our cohort may be attributable to cohort differences. Heikkinen et al. examined early-stage oral tongue cancer with lower overall event rates, while our cohort comprised of regionally advanced OCSCC across all oral cavity subsites, with a higher prevalence of adverse features and events. Such differences in disease stage, subsite distribution, and event frequency may have influenced the cutoff value. With inclusion of all subsites, generalizability of our findings to the full spectrum of OCSCC subsites is enhanced.

The concordance between independent studies, even with differences in tumor stage and subsite, provides the rationale for larger multi-center trials designed to further validate TIL prognostic thresholds. If confirmed, TIL status may be formally integrated into staging systems or risk stratification models, potentially influencing decisions on treatment. For example, high sTIL (IM) values might possibly identify patients who have a higher chance of benefit from adjuvant immunotherapy, while low counts could identify those in need of intensified surveillance [30]. Evaluating the immune response to malignancy is gaining attention due to the growing recognition of its prognostic significance and the expanding role of new immunotherapeutic agents across various cancer types [31,32,33]. However, this has always posed a challenge, especially in adoption to routine clinical practice, as many of the available techniques are technically complex, time-consuming, and associated with significant costs. In this setting, it is important to appreciate that TIL quantification according to this standardized method offers many practical advantages: it is inexpensive, rapid, reproducible, and performed on routine HE slides without necessitating costly immunohistochemistry or molecular assays. These features lend support to its incorporation into routine histopathological workflow for OCSCC, where a standardized TIL scoring protocol could be implemented alongside established histopathological assessments. Our study strengthens the existing data and provides a rationale for further investigation of the correlation between TILs and ENE, which has previously been assessed in a study on diagnostic biopsies; however, in resection specimens, tumor budding and DOI emerged as the stronger independent predictors [15].

Further research is warranted to define the functional mechanisms and phenotypic characteristics of these lymphocytes. Immunohistochemical and immunofluorescence analyses may enable discrimination between effector CD8+ T-cells, CD4+ subsets, FOXP3+ regulatory T-cells or memory lymphocyte populations. Spatial transcriptomics as well as gene expression profiling could expose activation signatures at the tumor–stroma interface. In addition, clonality studies and ex vivo assays might evaluate tumor-specific cytotoxic activity. Such studies will help elucidate the mechanism of action of TILs and evaluate their potential as biomarkers in future immunotherapy trials. 

We do acknowledge several limitations of this study. Retrospective and single-center design can induce selection bias and limit generalizability. Furthermore, while TIL assessment was performed using a standardized method, histopathological evaluation inherently carries a risk of interobserver variability. Therefore, the slides were independently assessed by two investigators blinded to patient identity and outcomes. However, some degree of subjectivity remains possible. Finally, our cohort size, although comparable to similar studies, limits the ability to perform subgroup analyses.

Despite these limitations, the strengths of this study include a review of uniformly treated, consecutive patients within a single, high-volume, tertiary referral center, as well as comprehensive inclusion of all AJCC-recommended clinicopathological features in the analyses, and application of a standardized, validated, and reproducible method for TIL assessment in the setting of regionally advanced oral cavity cancer.

## 5. Conclusions

Results of this study clearly demonstrated that higher sTIL (IM) is associated with significantly improved survival across all major endpoints (OS, DSS, DFS) in patients with regionally advanced OCSCC. This study adds to the existing literature by extending TIL evaluation to a cohort of consecutive, regionally advanced OCSCC of all oral cavity subsites, thereby providing broader insight into the prognostic significance of TILs in this heterogeneous tumor region.

Additionally, our dataset showed that lower sTIL (IM) was associated with the presence, but not the extent, of ENE, indicating that impaired host immune response may facilitate the development of ENE, without an impact on the quantitative burden of ENE.

Importantly, depth of invasion (DOI) also remained an independent predictor of outcome in the multivariable analyses, reaffirming its already well-established prognostic significance in OCSCC. These results demonstrate that sTIL (IM) provides complementary as well as independent prognostic information, beyond DOI and other adverse features. This announces the possibility of developing an encompassing prognostic algorithm that incorporates immune markers like sTIL (IM) to improve patient stratification. While this was beyond the scope of this retrospective single-center study, we believe our findings provide a rationale for future multi-center validation studies aimed at designing and testing integrative prognostic nomograms for OCSCC.

In the modern era of fast-evolving immunotherapy and personalized immuno-oncologic strategies, oral cancer immunology remains less explored with less established biomarker-based treatment algorithms and fewer immunotherapy options with modest benefit. The results of this study add to our collective knowledge about tumor microenvironment, immune profiling, and tumor–host interface in the context of oral cavity cancer. 

## Figures and Tables

**Table 1:** Optimal cutoff points for relevant variables.

Variable	Endpoint	Optimal Cutoff	*p*-Value
sTIL (TC) (%)	OS, DSS, DFS	15%	<0.001
sTIL (IM) (%)	OS, DSS, DFS	25%	<0.001
iTIL (TC) (%)	DSS	10%	0.042
iTIL (IM) (%)	OS, DSS	10%	0.010/0.013
iTIL (IM) (%)	DFS	20%	0.027
DOI (mm)	OS, DSS, DFS	9 mm	<0.001
Resection margin (mm)	OS, DSS, DFS	2.5 mm	0.012/0.015/0.009
Lymph node ratio (LNR)	OS, DSS, DFS	0.15	<0.001
Extranodal extension (mm)	OS, DSS, DFS	2 mm	<0.001

**Table 2:** Cohort clinical and histopathological characteristics and distribution of sTIL (IM).

Variables	Total *n* = 103	High sTIL (IM) (>25%)	Low sTIL (IM) (≤25%)	*p*-Value
Age (y)				0.576
≤60	66 (64.1%)	53 (80.3%)	13 (19.7%)	
60	37 (35.9%)	30 (81.1%)	7 (18.9%)	
Sex				0.442
Male	90 (87.4%)	71 (78.9%)	19 (21.1%)	
Female	13 (12.6%)	12 (92.3%)	1 (7.7%)	
Tumor site				0.391
Oral tongue	39 (37.9%)	31 (79.5%)	8 (20.5%)	
Floor of the mouth	34 (33.0%)	30 (88.2%)	4 (11.8%)	
Lower alveolar ridge	13 (12.6%)	8 (61.5%)	5 (38.5%)	
Retromolar trigone	12 (11.7%)	10 (83.3%)	2 (16.7%)	
Upper alveolar ridge	2 (1.9%)	2 (100%)	0 (0.0%)	
Buccal mucosa	2 (1.9%)	1 (50.0%)	1 (50.0%)	
Mucosal lip	1 (1.0%)	1 (100%)	0 (0.0%)	
Stage (AJCC) ^1^				0.288
III	10 (9.7%)	9 (90.0%)	1 (10.0%)	
IV.A	47 (45.6%)	40 (85.1%)	7 (14.9%)	
IV.B	46 (44.7%)	34 (73.9%)	12 (26.1%)	
Grade				0.427
I	43 (41.7%)	37 (86.0%)	6 (14.0%)	
II	44 (42.7%)	33 (75.0%)	11 (25.0%)	
III	16 (15.5%)	13 (81.2%)	3 (18.8%)	
PNI				0.287
Absent	39 (37.9%)	34 (87.2%)	5 (12.8%)	
Present	64 (62.1%)	49 (76.6%)	15 (23.4%)	
LVI				0.273
Absent	65 (63.1%)	55 (84.6%)	10 (15.4%)	
Present	38 (36.9%)	28 (73.7%)	10 (26.3%)	
WPOI-5				0.104
Absent	45 (43.7%)	40 (88.9%)	5 (11.1%)	
Present	58 (56.3%)	43 (74.1%)	15 (25.9%)	
ENE				0.142
Absent	52 (50.5%)	45 (86.5%)	7 (13.5%)	
Present	51 (49.5%)	38 (74.5%)	13 (25.5%)	

^1^ 8th Edition of AJCC Cancer Staging Manual [12].

**Table 3:** Univariable Cox regression analyses results, statistically significant variables.

Variables	OS HR (95% CI)	DSS HR (95% CI)	DFS HR (95% CI)
LVI			
Absent	1	1	1
Present	1.67 (1.06–2.63)	1.66 (1.03–2.70)	1.58 (1.01–2.47)
*p*	0.027	0.039	0.046
WPOI-5			
Absent	1	1	1
Present	1.67 (1.06–2.65)	1.67 (1.00–2.79)	1.75 (1.11–2.76)
*p*	0.028	0.048	0.016
iTIL (IM)			
Low	1	1	1
High	0.52 (0.31–0.86)	0.49 (0.27–0.87)	0.44 (0.21–0.93)
*p*	0.011	0.015	0.031
sTIL (TC)			
Low	1	1	1
High	0.28 (0.17–0.47)	0.32 (0.19–0.55)	0.30 (0.18–0.50)
*p*	<0.001	<0.001	<0.001
sTIL (IM)			
Low	1	1	1
High	0.12 (0.06–0.23)	0.14 (0.08–0.28)	0.13 (0.07–0.25)
*p*	<0.001	<0.001	<0.001
DOI			
Low (≤9 mm)	1	1	1
High (>9 mm)	2.95 (1.55–5.60)	3.73 (1.70–8.18)	3.20 (1.69–6.08)
*p*	<0.001	0.001	<0.001
Resection margin			
>2.5 mm	1	1	1
≤2.5 mm	1.91 (1.14–3.18)	1.83 (1.12–2.99)	1.95 (1.18–3.22)
*p*	0.014	0.016	0.009
LNR			
Low (≤0.15)	1	1	1
High (>0.15)	2.42 (1.48–3.94)	2.78 (1.64–4.72)	2.28 (1.41–3.71)
*p*	<0.001	<0.001	<0.001
ENE			
Micro (≤2 mm)	1	1	1
Macro (>2 mm)	3.41 (2.07–5.62)	3.14 (1.88–5.25)	3.43 (2.08–5.64)
*p*	<0.001	<0.001	<0.001

**Table 4:** Multivariable Cox regression analyses results, statistically significant variables.

Variable	OS HR (95% CI)	DSS HR (95% CI)	DFS HR (95% CI)
sTIL (IM)			
High (>25%)	1	1	1
Low (≤25%)	4.53 (1.57–13.10)	4.49 (1.48–13.58)	3.42 (1.17–9.99)
*p*	0.005	0.008	0.025
DOI (depth of invasion)			
Low (≤9 mm)	0.42 (0.21–0.85)	0.31 (0.13–0.73)	0.38 (0.19–0.78)
High (>9 mm)	1	1	1
*p*	0.015	0.007	0.009

## Data Availability

The raw data supporting the conclusions of this article will be made available by the authors on request.

## Abbreviations

The following abbreviations are used in this manuscript:

AJCC	American Joint Committee on Cancer
CI	Confidence interval
DFS	Disease-free survival
DOI	Depth of invasion
DSS	Disease-specific survival
ENE	Extranodal extension
HE	Hematoxylin–eosin
HR	Hazard ratio
iTIL (IM)	Intraepithelial tumor-infiltrating lymphocytes in the invasive margin
iTIL (TC)	Intraepithelial tumor-infiltrating lymphocytes in the tumor center
LNR	Lymph node ratio
LNY	Lymph node yield
LVI	Lymphovascular invasion
OCSCC	Oral cavity squamous cell carcinoma
OS	Overall survival
PNI	Perineural invasion
ROC	Receiver-operator characteristics
sTIL (IM)	Stromal tumor-infiltrating lymphocytes in the invasive margin
sTIL (TC)	Stromal tumor-infiltrating lymphocytes in the tumor center
TIL	Tumor-infiltrating lymphocytes
WHO	World Health Organization
WPOI-5	Worst pattern of invasion-5
